# 3D free-breathing late gadolinium enhancement 3T MRI: comparison with standard 2D Imaging

**DOI:** 10.1186/1532-429X-16-S1-P198

**Published:** 2014-01-16

**Authors:** Maurice Bizino, Qian Tao, Jacob Amersfoort, Rob J van der Geest, Hildo J Lamb

**Affiliations:** 1 LUMC, Leiden, Netherlands

## Background

Clinical cardiovascular magnetic resonance imaging (CMR) is routinely performed during multiple breath-holds in predefined 2D image orientations. 3D CMR is desirable, since it allows post-acquisition reformatting in any desired imaging plane. However, 3D CMR is hampered by long breath-hold duration, introducing patient discomfort and technical limitations. These limitations can be overcome by application of respiratory navigator techniques, for example to perform late gadolinium enhanced (LGE) CMR. The presently proposed 3D approach allows to: (1) perform 3D CMR during free-breathing; (2) increase image resolution; (3) average out heart rate variability by using a phase sensitive inversion recovery sequence (PSIR); (4) improve myocardial nulling, especially relevant at higher MR field strength such as 3T. Therefore, the purpose of the present study was to develop and clinically test 3D free-breathing PSIR LGE CMR using a respiratory navigator approach at 3T in comparison to a standard 2D breath-hold sequence.

## Methods

**Figure 1:**
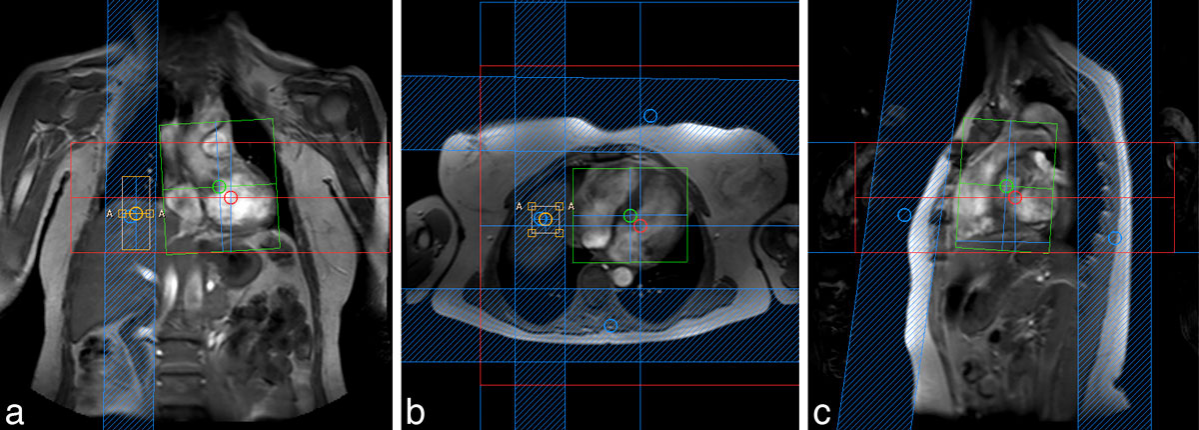
**a-c. Planning of the 3D navigator-gated PSIR sequence on coronal (a), transversal (b) and sagittal (c) planes**. The navigator box is placed on the right hemidiaphragm, the field of view covers the whole heart and rest slabs are used to prevent interference of navigator and subcutaneous adipose tissue.

In patients with a clinical indication for LGE imaging, both 3D and 2D PSIR sequences were acquired after administration of Dotarem (Guerbet) between May 2012 and June 2013. Figure 1 depicts planning of the 3D respiratory navigator gated sequence in coronal (a), transversal (b) and sagittal (c) planes, combined with rest slabs to suppress interference of the navigator and subcutaneous adipose tissue. Retrospectively, 26 patients showing LGE compatible with myocardial infarction, were selected for analysis (21 men; mean age ± standard deviation: 62.2 ± 7.8). After manual identification of endocardial and epicardial borders, myocardial scar tissue was identified and quantified semi-automatically in short axis slices. Myocardial scar tissue volume (ml), SNR, CNR and edge sharpness (ES) between healthy and scarred myocardium were quantified. Parameters were compared using a paired t-test.

## Results

**Figure 2:**
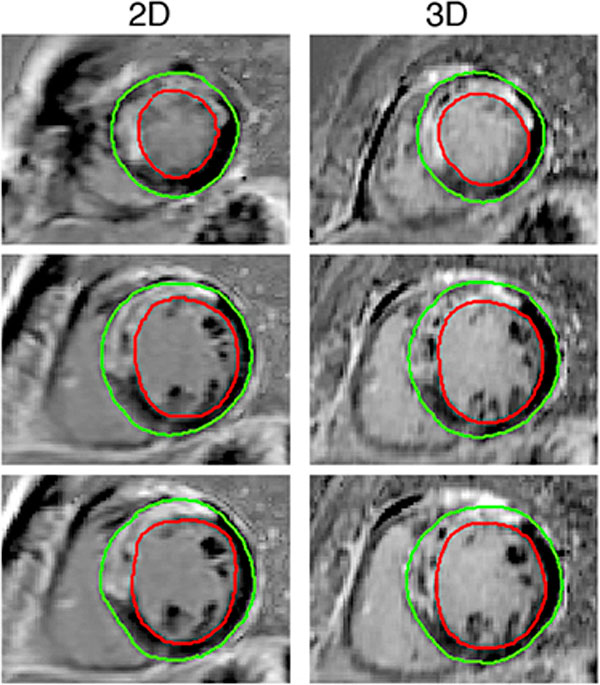
**Short-axis views of 2D and 3D sequences of a 58 year old man 2 weeks after an acute myocardial infarction with occlusion of the left anterior descending artery showing transmural delayed enhancement in septal and anterior segments**. Also note the necrotic core.

Figure 2 shows an example of short axis slices in 2D and analogous reconstructed slices from the 3D sequence. Myocardial scar tissue volume (3D: 27.11 ± 17.94; 2D: 29.50 ± 15.43 ml; p = 0.49), SNR (3D: 100.88 ± 31.97; 2D: 89.92 ± 18.57; p = 0.06) and CNR (3D: 3.96 ± 1.84; 2D: 3.55 ± 0.68; p = 0.25) were not significantly different between 3D and 2D sequences. However, ES was significantly higher for 3D CMR (3D: 0.051 ± 0.0072; 2D: 0.048 ± 0.0085; p = 0.019).

## Conclusions

Free-breathing 3D LGE CMR of myocardial scar tissue is feasible at 3T, with improved edge sharpness as compared to a standard 2D breath-hold PSIR sequence.

## Funding

No funding.

